# Muscle morphological adaptations to resistance training and sports participation in children and adolescents: a scoping review

**DOI:** 10.3389/fspor.2025.1646835

**Published:** 2025-10-08

**Authors:** Shota Enomoto, Nobuaki Tottori

**Affiliations:** ^1^ Institute for Promotion of Education and Campus Life, Okayama University, Okayama, Japan; ^2^ Graduate School of Education, Hyogo University of Teacher Education, Hyogo, Japan

**Keywords:** athlete, cross-sectional area, muscle thickness, muscle volume, muscle-strengthening activity, youth

## Abstract

**Introduction:**

This scoping review aimed to systematically map the existing literature on the effects of resistance training (RT) and sports participation on muscle morphology in children and adolescents.

**Methods:**

Herein, a literature search was conducted using three electronic databases: PubMed, Scopus, and Web of Science. The inclusion criteria were as follows: articles that were written in English, which used chronic RT or a combination of RT with other training methods, or investigated the effects of sports participation, and reported muscle morphology as an outcome.

**Results:**

This scoping review included 29 studies: 17 cross-sectional studies, 3 prospective observational studies, and 9 interventional studies. The following distribution was obtained after categorizing the included studies according to participant age: aged 6–11 years, 12 articles; aged 12–14 years, 10 articles; and aged 15–17 years, 10 articles. The designs of interventional studies included eight quasi-experimental parallel-group trials and a quasi-experimental crossover trial. However, none of the included interventional studies followed the CONSORT guidelines for conducting randomized controlled trials. Across the included studies, 14 different sports were analyzed for their effects on muscle morphology. Four studies combined players from various sports. In the included studies, 47 different muscles or muscle groups were examined. Our results identified unexplored muscles because our included studies did not examine the volume of lower leg muscles.

**Conclusion:**

Future research directions in this field, including experimental design and targeted muscles, are warranted.

## 1 Introduction

The World Health Organization recommended that children and adolescents should engage in at least 60 min of moderate-to-vigorous intensity physical activity daily throughout the week (1); additionally, muscle-strengthening activities were recommended at least three times per week (1). Several studies have investigated the effects of resistance training (RT) and sports participation, with recent reports highlighting their positive physical (2, 3) or mental (4, 5) benefits in children and adolescents. The effects of RT and sports participation include enhancements in strength (2, 6), fundamental movement skills (7, 8), and academic performance (9, 10), as well as a particularly noteworthy alteration in muscle morphology.

Evidence suggests that an individual's lean mass is established before adolescence and persists into adulthood (11), emphasizing the importance of increasing muscle mass during childhood for better health in later life. Furthermore, greater muscle size in children is reportedly associated with superior sprint (12–15) and agility (13) performances. Therefore, it is important to elucidate the trainability of muscle morphology to RT and sports participation during childhood.

Studies on adults have shown that RT (16) and sports participation (17) induce muscle hypertrophy. In contrast, a previous study reported that in prepubertal children (18), RT did not lead to muscle hypertrophy that exceeded natural growth. However, another study contradicting this observation suggested that prepubertal children may experience muscle hypertrophy beyond their natural growth when engaged in RT (19). Similarly, findings regarding sports participation remain inconsistent. Hoshikawa et al. (20) showed that adolescent soccer players exhibited a larger cross-sectional area (CSA) of the psoas major than age-matched non-athletes. In contrast, another study reported no significant differences in CSA of rectus femoris between pre- or early-pubertal swimmers, gymnasts, and age-matched non-athletes (21). The conflicting findings regarding the effects of RT and sports participation in studies on childhood may be attributed to differences in factors across studies, such as participant age, intervention and study duration, training volume, load, and the type of sport examined. Therefore, it is essential to synthesize the available evidence to better understand the current state of knowledge in this field and identify directions for future studies.

Several review articles have synthesized the morphological adaptations of muscles to RT and sports participation in children and adolescents (22–24). Legerlotz et al. (22) reviewed physiological adaptations to RT in young athletes. Tumkur Anil Kumar et al. (24) reviewed the effect of RT on the muscle-tendon unit in youth. However, since these reviews were narrative reviews, previous studies were not systematically included. Sánchez Pastor et al. (23) conducted a systematic review that focused exclusively on prepubertal children.

Therefore, this study aimed to systematically map existing literature on the effects of RT and sports participation on muscle morphology in children and adolescents. Specifically, our review evaluated the types of studies that investigated the effects of RT and sports participation on muscle morphological adaptation in children and adolescents, the chronological and biological age and sex groups the studies focused on, the muscles that were examined in these studies, and the existing gaps in evidence in this field.

## 2 Materials and methods

### 2.1 Design

To systematically synthesize existing evidence, several types of reviews are employed based on the purpose (25), such as systematic reviews or scoping reviews (25). We conducted a scoping review based on the aim of this study. This scoping review was conducted in accordance with the Preferred Reporting Items for Systematic Reviews and Meta-Analyses for Scoping Reviews (26) and followed the methodological framework of Arksey and O’Mallay (27).

### 2.2 Information sources and search strategies

The literature search was conducted in November 2024 using three electronic databases: PubMed, Scopus, and Web of Science. The “Population, Concept, and Context” approach was used to design the eligibility criteria, referring to children and adolescents (<18 years old at baseline) without diseases, any intervention of RT or exposure to any sports activity, and evaluation of muscle morphology. Database searches were conducted using a combination of terms such as “child*”, “preadolescen*”, “adolescen*”, “junior”, “resistance”, “exercise”, “sport*”, “cross-sectional area”, “muscle thickness”, “muscle hypertrophy”, and “muscle morphology”. These terms are provided as examples and do not represent the full list of search keywords used. The full search strategy for each database is shown in the Supplementary Tables.

### 2.3 Study selection

Among studies identified in the literature search, duplicates were removed using EndNote (Endnote 20.6; Clarivate Analytics, PA, USA). In the first screening step, eligible studies were independently selected by two authors based on titles and abstracts. In the second screening step, eligible studies were independently selected by two authors based on their full text. After the completion of each step, the authors discussed discrepancies in decisions, which were resolved through consensus.

### 2.4 Eligibility criteria

Eligibility criteria to screen studies in this study were as follows: (1) published in peer-reviewed journals, (2) included healthy or typically developing children, (3) studies on humans, (4) reported muscle morphology as an outcome, (5) included children aged <18 years old at baseline, (6) used chronic RT or combinations of RT with other training or investigated the effects of sports participation, (7) had a control group to demonstrate the effects of RT or sports participation, and (8) were written in English. No date restriction was imposed for the search. Studies were excluded if they (1) involved only children with diseases, (2) reported only body mass index or circumference or fat-free mass as indicators of muscle mass or size, and (3) were review articles.

### 2.5 Summarizing the findings

Microsoft Excel was used to calculate the descriptive statistics of the data extracted from the included articles, which were summarized and grouped based on categories.

### 2.6 Data charting

Two authors developed a data-charting form to address the purpose of this study. The data were extracted and then charted using Microsoft Excel. The following information was collected from all included studies: publication year, country of the first author, study design, sample size, sex, age, biological age, competition history and level of participants, targeted muscle, measurement equipment, whether an *a priori* power analysis was conducted, and outcomes related to muscle morphology (i.e., muscle thickness, CSA, volume, fascicle length, and pennation angle). For interventional studies, the following information was additionally collected: intensity, frequency, duration, and volume of the training program. The following information was additionally collected for observational studies: number of measurements and study duration.

## 3 Results

**Figure 1:**
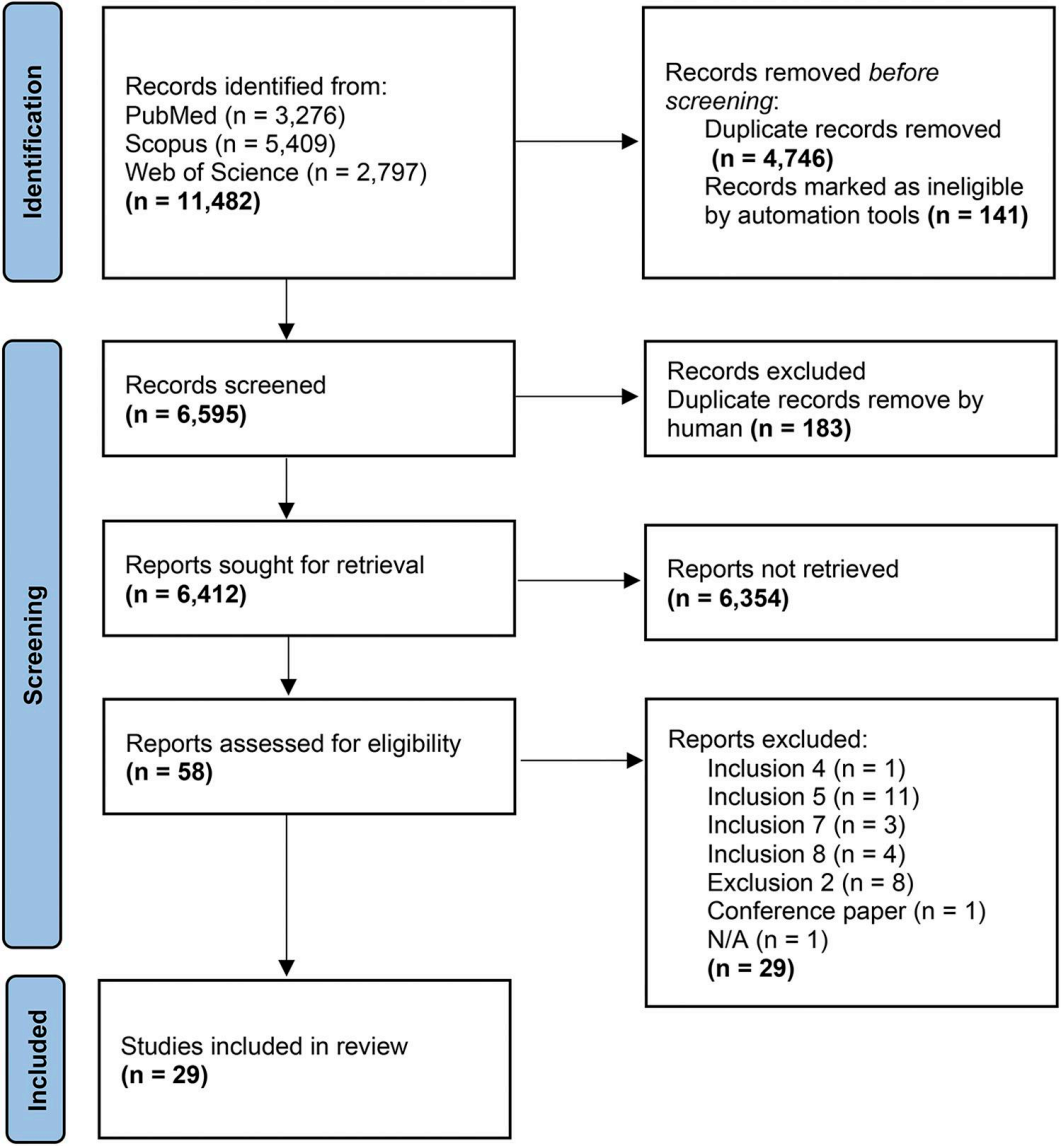
PRISMA flow diagram of the literature search.

Among 11,482 articles initially identified, 29 were considered eligible for inclusion after screening (Figure 1).

### 3.1 Study description

**Figure 2:**
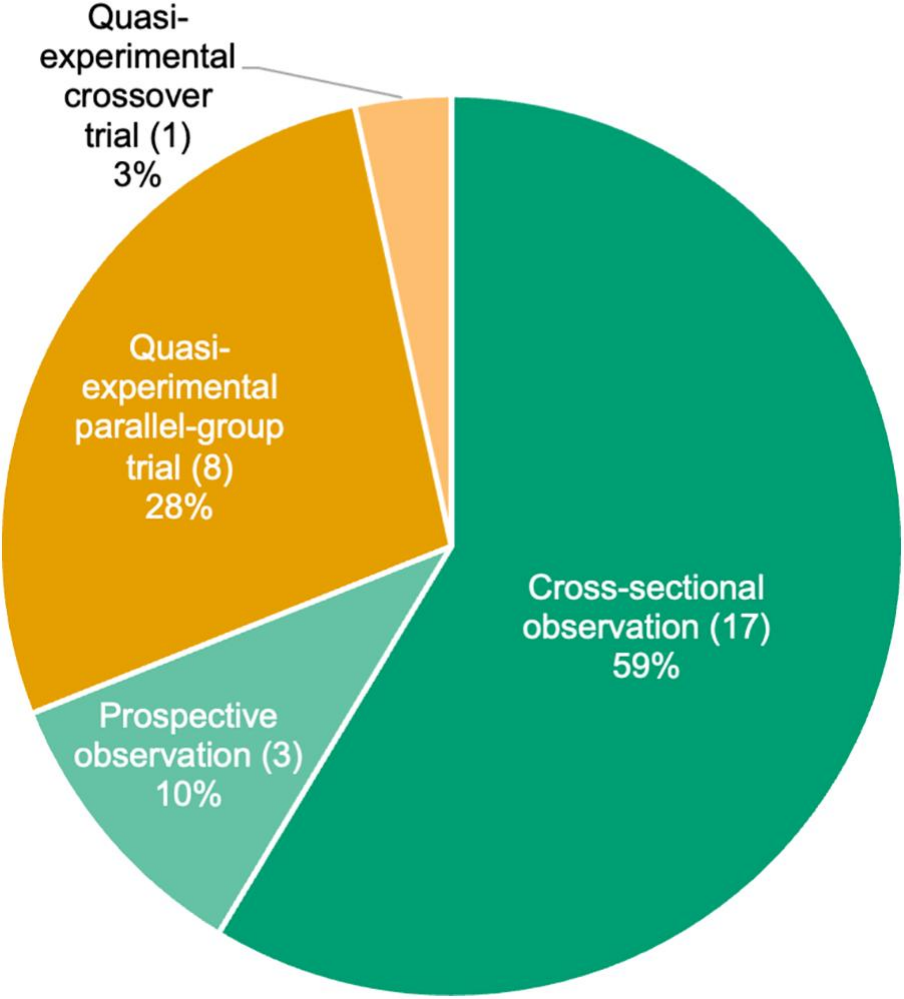
Number and percentage of studies by study design.

**Figure 3:**
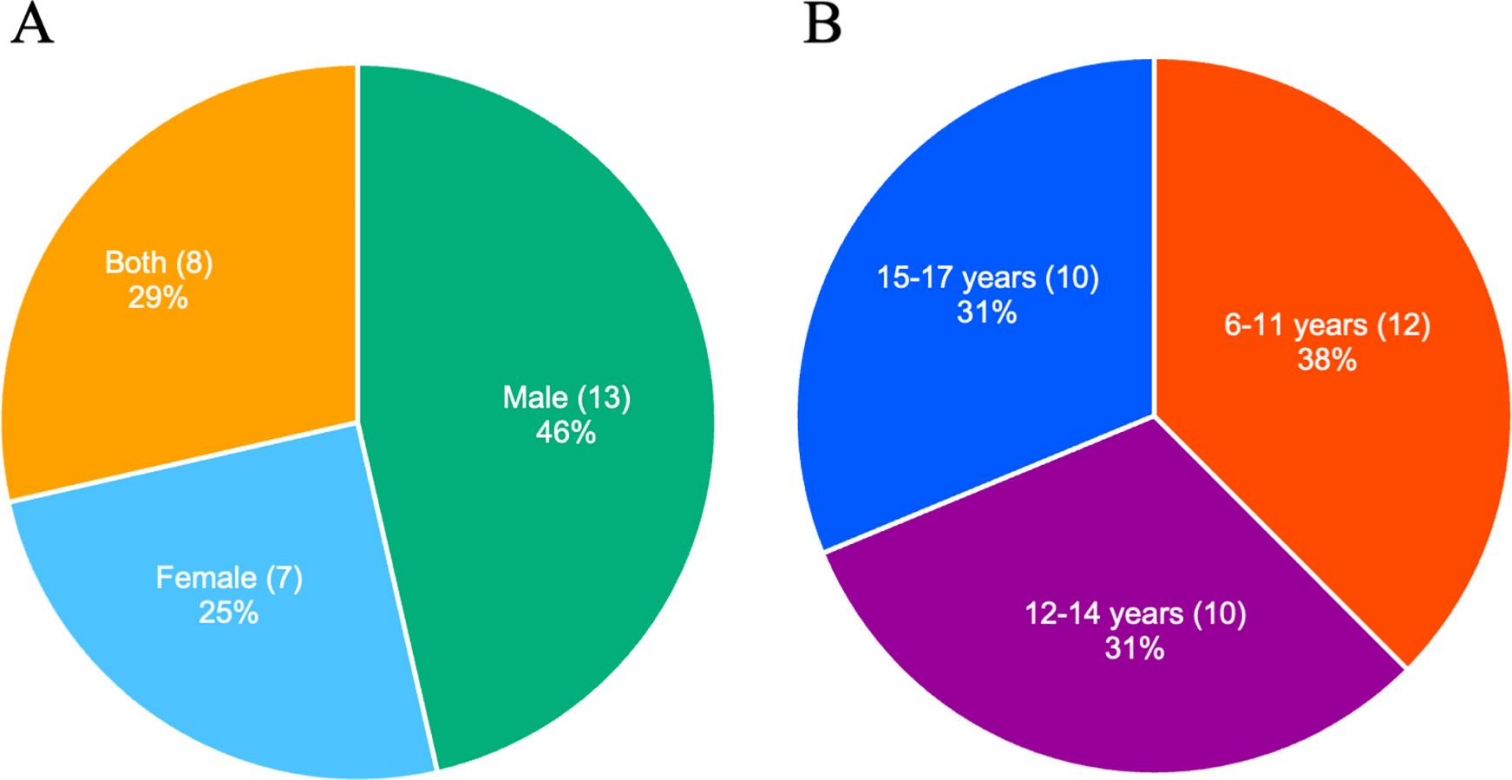
Number and percentage of studies by sex **(A)** and age categories **(B)**. A study that did not report the participant's sex is excluded **(A)**.

The included studies were conducted in Japan, Germany, Spain, Australia, the United States, Canada, Finland, France, the United Kingdom, and Colombia (eight, six, four, three, two, two, one, one, one, and one articles, respectively) (Table 1). We included articles published in the 1990s or later, with 5, 2, 16, and 6 studies conducted in the 1990s, 2000s, 2010s, and 2020s, respectively. The study design included 20 observational studies (17 cross-sectional and 3 prospective studies) and 9 interventional studies (Table 1, Figure 2). Among the studies, 13, 7, and 8 included male, female, and both sexes as participants, respectively; one study (50) did not report the sex of the subjects (Figure 3). The age ranges were 6–11, 12–14, and 15–17 years in 12, 10, and 10 studies, respectively (Figure 3). Six studies performed a power analysis (34–36, 43, 45, 46).

**Table 1:** Characteristics of the included studies (*n* = 29).

No.	Author	Year	Study design	Country of origin	Activity	Sample size	Age (years)	Biological age (methods)	Outcome (Measurement)	Targeted muscle
1	Peltonen et al. (44)	1997	Cross-sectional	FI	Sports: figure skating, gymnastics, ballet dance	Sports: 49 (F) Control: 17 (F)	Sports: figure skating; 14.6 ± 0.7, gymnastics; 14.7 ± 1.0, ballet dance; 14.8 ± 0.6 (mean ± SD) Control: 14.9 ± 0.4 (mean ± SD)	N/A	CSA (MRI)	Psoas, multifidus + erector spinae
2	Greene et al. (36)	2005	Cross-sectional	AU	Middle-distance running	Middle-distance running: 20 (F) Control: 20 (F)	Middle-distance running: 15.9 ± 1.6 (mean ± SD) Control: 16.0 ± 1.8 (mean ± SD)	N/A (Tanner)	CSA (MRI)	Lower leg: extensors, flexors
3	Kanehisa et al. (40)	2005	Cross-sectional	JP	Weightlifting	Weightlifting: 7 (M) Control: 13 (M)	Weightlifting: 15.1 ± 0.3 (mean ± SD) Control: 15.1 ± 0.3 (mean ± SD)	Weightlifting: 16.4 ± 0.7 (mean ± SD; TW2) Control: 16.3 ± 0.6 (mean ± SD; TW2)	Thickness (Ultrasound)	Anterior forearm, anterior upper arm, posterior upper arm, chest, abdomen, back, anterior thigh, posterior thigh, anterior lower leg, posterior lower leg
4	Hoshikawa et al. (39)	2010	Cross-sectional	JP	Soccer Volleyball Rowing Karate Sumo Sprinting Throwing	Soccer: 32 (M) Volleyball: 21 (M) Rowing: 29 (M) Karate: 18 (M) Sumo: 15 (M) Sprinting: 22 (M) Throwing: 16 (M) Control: 20 (M)	Soccer: 17.3 ± 0.5 (mean ± SD) Volleyball: 17.2 ± 0.5 (mean ± SD) Rowing: 17.2 ± 0.5 (mean ± SD) Karate: 17.3 ± 0.5 (mean ± SD) Sumo: 17.2 ± 0.6 (mean ± SD) Sprinting: 17.3 ± 0.5 (mean ± SD) Throwing: 17.3 ± 0.6 (mean ± SD) Control: 17.2 ± 0.4 (mean ± SD)	N/A	CSA (MRI)	Total thigh, quadriceps femoris, hamstrings
5	Hoshikawa et al. (38)	2011	Cross-sectional	JP	Sprinting Jumping Throwing	Sprinting: 61 (M = 29, F = 32) Jumping: 50 (M = 28, F = 22) Throwing: 33 (M = 18, F = 15) Control: 40 (M = 20, F = 20)	Sprinting: M; 17.3 ± 0.5, F; 17.2 ± 0.6 (mean ± SD) Jumping: M; 17.3 ± 0.4, F; 17.1 ± 0.7 (mean ± SD) Throwing: M; 17.2 ± 0.5, F; 17.3 ± 0.6 (mean ± SD) Control: M; 17.3 ± 0.5, F; 17.1 ± 0.5 (mean ± SD)	N/A	CSA (MRI)	Quadriceps femoris, hamstrings, adductors, psoas major
6	Mitchell et al. (21)	2011	Cross-sectional	CA	Endurance: swimming Power: gymnastics	Endurance: 12 (M) Power: 9 (M) Control: 18 (M)	Endurance: 10.7 ± 0.7 (mean ± SD) Power: 9.3 ± 1.3 (mean ± SD) Control: 9.9 ± 1.3 (mean ± SD)	Endurance: −3.2 ± 0.6 (mean ± SD; years from PHV) Power: −3.7 ± 0.9 (mean ± SD; years from PHV) Control: −3.9 ± 0.9 (mean ± SD; years from PHV)	Estimated CSA (Ultrasound)	Rectus femoris, biceps femoris
7	Burt et al. (29)	2012	Cross-sectional	AU	Gymnastics	High-training gymnastics: 30 (F) Low-training gymnastics: 29 (F) Control: 29 (F)	High-training gymnastics: 8.9 (mean), 6–11 (range) Low-training gymnastics: 8.3 (mean), 6–11 (range) Control: 8.6 (mean), 6–11 (range)	Stage I-II (Tanner Breast and Pubic hair)	CSA (CT)	Total forearm
8	Hoshikawa et al. (20)	2012	Cross-sectional	JP	Soccer	Late adolescent soccer: 27 (M) Late adolescent control: 20 (M) Early adolescent soccer: 22 (M) Early adolescent control: 11 (M)	Late adolescent soccer: 16.1–17.9 (range) Late adolescent control: 16.0–17.7 (range) Early adolescent soccer: 12.8–13.6 (range) Early adolescent control: 12.6–13.5 (range)	N/A	CSA (MRI)	Psoas major
9	Sanchis-Moysi et al. (49)	2012	Cross-sectional	ES	Tennis	Tennis: 7 (M) Control: 7 (M)	Tennis: 11.0 ± 0.8 (mean ± SD) Control: 11.0 ± 0.8 (mean ± SD)	Stage I-II (Tanner)	Volume (MRI)	Upper arm: deltoid, triceps, flexors, total upper arm Forearm: flexors, extensors, supinator, mobile wad, total forearm
10	Sanchis-Moysi et al. (47)	2016	Cross-sectional	ES	Tennis	Tennis: 6 (M) Control: 6 (M)	Tennis: 11.0 ± 0.9 (mean ± SD) Control: 10.7 ± 0.5 (mean ± SD)	Stage I-II (Tanner)	Volume (MRI)	Pectoralis
11	Mersmann et al. (41)	2017	Cross-sectional	DE	Volleyball	Volleyball: 21 (M = 12, F = 9) Control: 24 (M = 12, F = 12)	Volleyball: M; 16.8 ± 1.0, F; 16.7 ± 0.9 (mean ± SD) Control: M; 16.8 ± 1.1, F; 16.6 ± 0.9 (mean ± SD)	N/A	Thickness, pennation angle, fascicle length (Ultrasound)	Vastus lateralis
12	Sanchis-Moysi et al. (48)	2017	Cross-sectional	ES	Tennis	Tennis: 7 (M) Control: 10 (M)	Tennis: 11.0 ± 0.8 (mean ± SD) Control: 11.0 ± 0.7 (mean ± SD)	Stage I-II (Tanner)	Volume (MRI)	Rectus abdominis, obliques + transversus abdominis, quadratus lumborum, paravertebralis (longissimus thoracis, iliocostalis lumborum, multifidus, spinalis thoracis), iliopsoas, gluteus
13	Charcharis et al. (30)	2019	Cross-sectional	DE	Sports: american football, volleyball, handball, basketball, judo, kick-boxing, fencing, gymnastics, dancing, hockey, vaulting, track and field, acrobatics, decathlon (exclude endurance sports)	Late adolescent sports: 14 (M) Late adolescent control: 13 (M) Early adolescent sports: 15 (M) Early adolescent control: 14 (M)	Late adolescent sports: 17.2 ± 0.8 (mean ± SD) Late adolescent control: 17.3 ± 0.8 (mean ± SD) Early adolescent sports: 13.0 ± 0.8 (mean ± SD) Early adolescent control: 12.8 ± 0.6 (mean ± SD)	N/A	Thickness, pennation angle, fascicle length (Ultrasound)	Vastus lateralis
14	Gomez-Bruton et al. (34)	2019	Cross-sectional	ES	Swimming	Swimming: 65 (M = 31, F = 34) Control: 119 (M = 68, F = 51)	Swimming: M; 15.1 ± 1.5, F; 13.9 ± 1.9 (mean ± SD) Control: M; 14.9 ± 2.3, F; 14.2 ± 2.3 (mean ± SD)	Stage I-V (Tanner)	CSA (CT)	Total forearm
15	Giraldo García et al. (33)	2020	Cross-sectional	CO	Soccer Multisports: volleyball, basketball, swimming, gymnastics, cheerleading	Soccer: 82 (M = 81, F = 1) Multisports: 58 (M = 15, F = 43) Control: 44 (M = 13, F = 31)	Soccer: M; 8.9 ± 1.16 (mean ± SD), F; 10.7 Multisports: M; 9.2 ± 1.04, F; 9.1 ± 1.07 (mean ± SD) Control: M; 9.8 ± 0.55, F; 9.8 ± 0.55 (mean ± SD)	Stage I (Tanner)	Thickness, pennation angle (Ultrasound)	Thickness: anterior thigh, lateral thigh, rectus femoris, vastus lateralis, vastus intermedius Pennation angle: rectus femoris, vastus lateralis
16	Mersmann et al. (42)	2020	Cross-sectional	DE	Sports: handball, basketball	Sports: 14 (M) Control: 10 (M)	Sports: 13.9 ± 0.5 Control: 13.4 ± 1.0	N/A	Volume, ACSA, PCSA (MRI) Fascicle length, pennation angle (Ultrasound)	Vastus lateralis
17	Pentidis et al. (45)	2020	Cross-sectional	DE	Gymnastics (artistic gymnastics)	Artistic gymnastics: 21 (M = 6, F = 15) Control: 11 (M = 5, F = 6)	Artistic gymnastics: 9.2 ± 1.7 (mean ± SD) Control: 9.0 ± 1.7 (mean ± SD)	Stage I-II (Tanner)	Thickness, pennation angle (Ultrasound)	Medial gastrocnemius
18	Hoshikawa et al. (37)	2013	Prospective	JP	Soccer	Soccer: 24 (M) Control: 11 (M)	Soccer: 12.8 ± 0.3 (mean ± SD) Control: 12.7 ± 0.2 (mean ± SD)	N/A	CSA (MRI)	Total thigh, quadriceps femoris, hamstrings
19	Pentidis et al. (46)	2021	Prospective	DE	Gymnastics (artistic gymnastics)	Artistic gymnastics: 21 (M = 6, F = 15) Control: 11 (M = 5, F = 6)	Artistic gymnastics: 9.2 ± 1.7 (mean ± SD) Control: 9.0 ± 1.7 (mean ± SD)	Stage I-II (Tanner)	Thickness, pennation angle, fascicle length (Ultrasound)	Medial gastrocnemius
20	Birat et al. (28)	2024	Prospective	FR	Triathlon	Triathlon: 23 (M) Control: 15 (M)	Triathlon: 13.9 ± 0.6 (mean ± SD) Control: 14.0 ± 0.6 (mean ± SD)	Triathlon: −0.1 ± 0.9 (mean ± SD; years from PHV) Control: −0.1 ± 0.7 (mean ± SD; years from PHV)	Thickness, pennation angle, fascicle length (Ultrasound)	Vastus lateralis, rectus femoris
21	Ramsay et al. (18)	1990	Quasi-experimental parallel-group trial	CA	Resistance training	Intervention: 13 (M) Control: 13 (M)	Total: 9–11 (range)	Stage I (Tanner)	CSA (CT)	Biceps brachii + brachialis, quadriceps femoris
22	Fukunaga et al. (19)	1992	Quasi-experimental parallel-group trial	JP	Resistance training	Intervention 1st grade: M = 8, F = 7 3rd grade: M = 10, F = 7 5th grade: M = 10, F = 10 Control 1st grade: M = 8, F = 6 3rd grade: M = 8, F = 9 5th grade: M = 8, F = 8	Intervention (mean ± SD) 1st grade: M; 6.9 ± 0.3, F; 7.0 ± 0.3 3rd grade: M; 9.0 ± 0.3, F; 9.0 ± 0.4 5th grade: M; 11.0 ± 0.3, F; 10.9 ± 0.3 Control (mean ± SD) 1st grade: M; 7.0 ± 0.3, F; 7.0 ± 0.3 3rd grade: M; 9.0 ± 0.2, F; 9.0 ± 0.3 5th grade: M; 11.1 ± 0.2, F; 11.2 ± 0.2	Intervention (mean ± SD; TW2) 1st grade: M; 6.2 ± 0.5, F; 6.5 ± 1.6 3rd grade: M; 8.1 ± 1.1, F; 8.6 ± 0.8 5th grade: M; 10.7 ± 2.0, F; 10.7 ± 0.9 Control (mean ± SD; TW2) 1st grade: M; 6.4 ± 0.9, F; 6.0 ± 1.1 3rd grade: M; 8.8 ± 0.6, F; 8.3 ± 0.9 5th grade: M; 10.8 ± 1.0, F; 10.9 ± 0.7	CSA (Ultrasound)	Upper arm: total upper arm, extensors, flexors, biceps brachii, brachialis
23	Eliakim et al. (31)	1996	Quasi-experimental parallel-group trial	US	Multiple exercise and sports	Intervention: 22 (F) Control: 22 (F)	Total: 15–17 (range)	Stage V (Tanner)	Volume (MRI)	Total thigh
24	Eliakim et al. (32)	1997	Quasi-experimental parallel-group trial	US	Multiple exercise and sports	Intervention: 22 (F) Control: 22 (F)	Total: 15–17 (range)	95% of the participants; Stage V (Tanner)	Volume (MRI)	Total thigh
25	Granacher et al. (35)	2011	Quasi-experimental parallel-group trial	DE	Resistance training	Intervention: 17(M = 8, F = 9) Control: 15 (M = 10, F = 5)	Intervention: 8.6 ± 0.5 (mean ± SD) Control: 8.7 ± 0.5 (mean ± SD)	Stage I (Tanner)	CSA (MRI)	Quadriceps femoris
26	Takai et al. (51)	2013	Quasi-experimental parallel-group trial	JP	Resistance training	Intervention: 36 (M) Control: 58 (M)	Intervention: 13.6 ± 0.6 (mean ± SD) Control: 13.8 ± 0.5 (mean ± SD)	Intervention: Stage 3.5 ± 1.4 (mean ± SD; Tanner) Control: Stage 3.6 ± 1.2 (mean ± SD; Tanner)	Thickness (Ultrasound)	Anterior thigh
27	Yoshimoto et al. (52)	2016	Quasi-experimental parallel-group trial	JP	Resistance training	Intervention: 27 (F) Control: 20 (F)	Intervention: 13.8 ± 0.6 (mean ± SD) Control: 13.8 ± 0.5 (mean ± SD)	Intervention: Stage 3.9 ± 0.9 (mean ± SD; Tanner) Control: Stage 3.7 ± 0.9 (mean ± SD; Tanner)	Thickness (Ultrasound)	Anterior thigh
28	Secomb et al. (50)	2017	Quasi-experimental crossover trial	AU	Resistance training (Intervention 1), Plyometrics & gymnastics (Intervention 2)	Intervention1: 8 (N/A) Intervention2: 8 (N/A) Control: 8 (N/A)	Total: 14.8 ± 1.8 (mean ± SD)	N/A	Thickness, pennation angle, fascicle length (Ultrasound)	Vastus lateralis, lateral gastrocnemius
29	Moeskops et al. (43)	2024	Quasi-experimental parallel-group trial	GB	Gymnastics & neuromuscular training (Intervention 1), Gymnastics only (Intervention 2)	Intervention 1: 15 (F) Intervention 2: 10 (F) Control: 12 (F)	Intervention 1: 9.4 ± 1.8 (mean ± SD) Intervention 2: 9.9 ± 1.2 (mean ± SD) Control: 8.7 ± 1.6 (mean ± SD)	Intervention 1: 80.95 ± 7.29% (mean ± SD; %PAH) Intervention 2: 83.57 ± 5.18% (mean ± SD; %PAH) Control: 79.00 ± 8.05% (mean ± SD; %PAH)	Thickness, pennation angle, fascicle length (Ultrasound)	Medial gastrocnemius

AU, Australia; CA, Canada; CO, Colombia; DE, Germany; ES, Spain; FI, Finland; FR, France; GB, United Kingdom; JP, Japan; US, United States; M, male; F, female; TW2, Tanner-Whitehouse II methods; PHV, peak height velocity; %PAH, % predicted adult height; MRI, magnetic resonance imaging; CT, computerized tomography; CSA, cross-sectional area.

**Figure 4:**
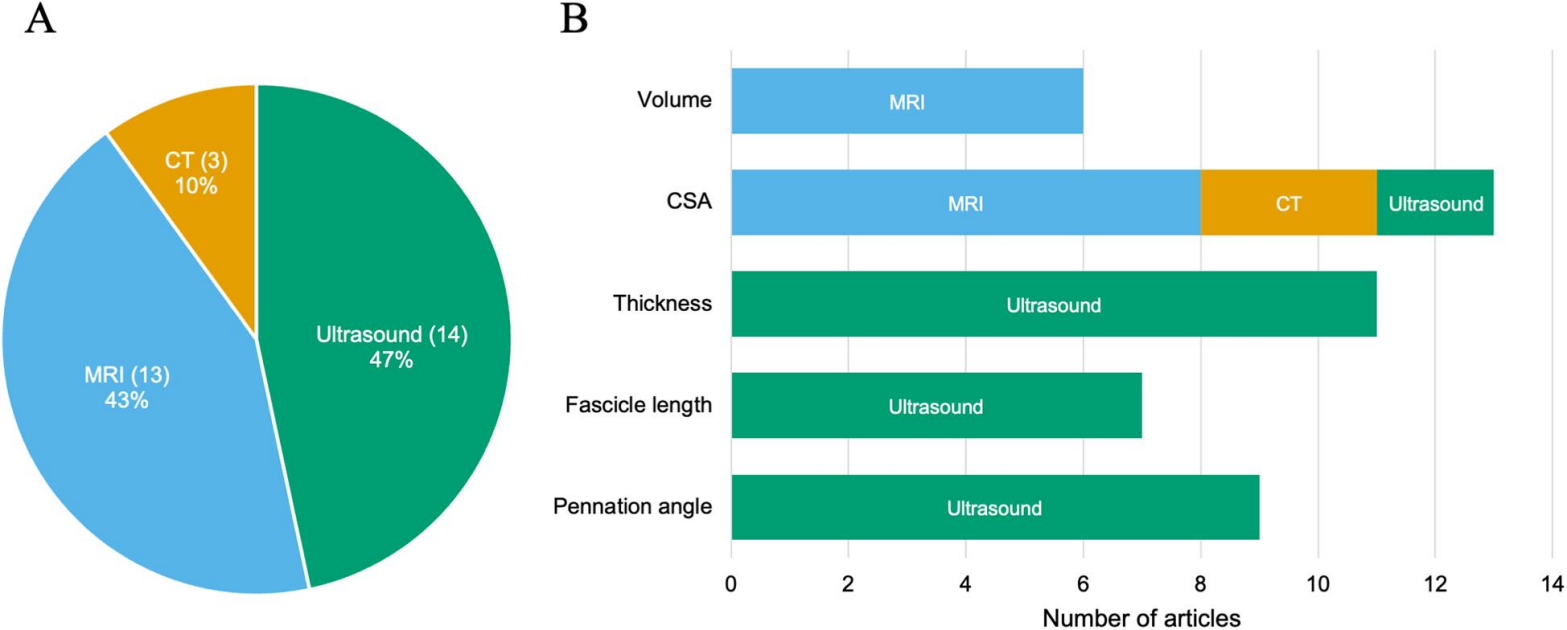
Number and percentage of studies by assessment methods **(A)** and variables **(B)**, MRI, magnetic resonance imaging; CT, computerized tomography; CSA, cross-sectional area.

Biological age was assessed in 20 articles, including one (36) that did not report results (Table 1). Assessments of maturity status included Tanner stage (15 articles), predicted peak height velocity (two articles), Tanner-Whitehouse II methods (two articles), and percentage predicted adult height (one article) (Table 1). Muscle morphology was assessed using magnetic resonance imaging (13 articles), ultrasonography (14 articles), and computed tomography (3 articles) (Figure 4). The measured variables were muscle volume, CSA, thickness, fascicle length, and pennation angle. The evaluation targets of the muscles for size are listed in Table 2.

**Table 2:** Targeted muscles and muscle groups for size evaluation.

Measurement	Forearm	Upper arm	Trunk	Thigh	Lower leg
Volume	Total forearm Extensors Flexors Mobile wad Supinator	Total upper arm Deltoid Flexors Triceps	Gluteus Iliopsoas Paravertebralis Pectoralis Obliques Quadratus lumborum Rectus abdominis Transversus abdominis	Total thigh Vastus lateralis	
CSA	Total forearm	Total upper arm Biceps brachii Brachialis Extensors Flexors	Erector spinae Multifidus Psoas Psoas major	Total thigh Adductors Biceps femoris Hamstrings Quadriceps femoris Rectus femoris Vastus lateralis	Extensors Flexors
Thickness	Anterior	Anterior Posterior	Abdomen Back Chest	Anterior Lateral Posterior Rectus femoris Vastus intermedius Vastus lateralis	Anterior Lateral gastrocnemius Medial gastrocnemius Posterior

CSA, cross-sectional area. The underline indicates muscles evaluated in interventional studies.

### 3.2 Observational studies

Total sample sizes ranged from 12 to 184 (Table 1). Targeted activities included middle-distance running, weightlifting, soccer, volleyball, rowing, karate, sumo, sprinting, throwing, jumping, swimming, tennis, gymnastics, and triathlon. Four studies combined players from various sports. Four articles focused on the sex differences in muscle morphology due to participation in sports (33, 34, 38, 41). Two articles focused on age differences in muscle morphology due to participation in sports and recruited middle- and late-adolescent boys (20, 30).

Regarding prospective studies, the total sample sizes ranged from 32 to 38. Two studies recruited males, and one study recruited participants of both sexes. The durations of study were 6 (37), 9 (28), and 12 (46) months. One article assessed the outcome measures more than three times, at three-month intervals (46).

### 3.3 Interventional studies

The designs of interventional studies included eight quasi-experimental parallel-group trials and a quasi-experimental crossover trial (Table 1, Figure 2). No study followed the CONSORT guidelines for conducting randomized controlled trials (RCTs). The sample size per group ranged from 6 to 58. The age ranges were 6–11, 12–14, and 15–17 years in 4, 3, and 2 studies, respectively. Among the nine studies, five involved participants with no athletic background (18, 19, 31, 32, 35), two involved participants engaged in extracurricular activities at a regional competitive level (51, 52), one involved surfing athletes (50), and one involved gymnasts (43). The intervention periods were from 5 weeks to 10 months. The activities of the intervention group included RT, multiple exercise and sports, gymnastics, plyometrics, and neuromuscular exercise (Table 3). The frequency of intervention was two to six times per week. One article focused on the differences in training effects between sexes or grades in school (19). Volume was only evaluated for the total thigh muscle group (Table 2).

**Table 3:** Characteristics of the included interventional studies (*n* = 9).

No.	Author	Intervention type	Duration	Frequency (days per week)	Total days
21	Ramsay et al. (18)	Resistance training (preacher arm curl, double leg extension, leg press, bench press, behind the neck pulldown, sit-ups or trunk curls)	20 weeks	3	60
22	Fukunaga et al. (19)	Resistance training (isometric training of elbow flexion)	12 weeks	3	36
23	Eliakim et al. (31)	Multiple exercise and sports [running, aerobic dance, competitive sports (e.g., basketball), occasional weightlifting]	5 weeks	5	25
24	Eliakim et al. (32)	Multiple exercise and sports [running, aerobic dance, competitive sports (e.g., basketball), occasional weightlifting]	5 weeks	N/A	N/A
25	Granacher et al. (35)	Resistance training (weight-machine based high intensity strength training; leg-press, knee extension/flexion, seated calf-raise, hip abduction/adduction, core exercise)	10 weeks	2	20
26	Takai et al. (51)	Resistance training (body-mass based squat)	8 weeks	4–6	45
27	Yoshimoto et al. (52)	Resistance training (body-mass based squat)	8 weeks	4–6	45
28	Secomb et al. (50)	Resistance training	7 weeks	2	14
		Plyometrics & gymnastics	7 weeks	2	14
29	Moeskops et al. (43)	Competitive gymnastics training &	10 months	2–5	N/A
		neuromuscular training		2	
		Competitive gymnastics training	10 months	2–5	NA

## 4 Discussion

This scoping review aimed to systematically map the existing evidence on the effects of RT and sports participation on muscle morphology in children and adolescents. This study included 29 articles and clarified the current state of research and gaps in the relevant literature.

### 4.1 Study design

This study included interventional and observational studies. Nine studies examined the effects of RT or sports activities compared with a control group. The designs of these studies included eight quasi-experimental parallel-group trials and a quasi-experimental crossover trial. However, none of these studies followed the CONSORT guidelines for RCTs, which are the primary standard for determining relationships between interventions and outcomes. In studies involving adults, numerous RCTs have been conducted on the effects of RT on muscle hypertrophy (53), and systematic reviews synthesizing these findings have contributed to a growing body of high-level evidence on the effects of RT on muscle morphology (54). Our findings not only emphasize the scarcity of studies on children and adolescents but also highlight the low quality of existing evidence compared with studies on adults. In contrast to research involving adult populations, interventions are often implemented at the class level during regular classes of children and adolescents, making it difficult to randomly assign individual participants. In this review, most of the interventional studies stated that they conducted interventions in school settings (31, 32, 35, 51, 52). Moreover, the studies included in this review were non-RCTs with relatively small sample sizes, precluding the implementation of intention-to-treat analysis. By comparison, both RCTs (55) and cluster RCTs (56) have been implemented as part of efforts to strengthen the evidence in other research fields. Future studies should consider adopting designs such as that of cluster RCTs, for example by utilizing after-school programs or community-based settings as intervention platforms.

Additionally, 20 studies examined the effects of sports participation, including 17 cross-sectional studies and three prospective observational studies. Time-course data on muscle morphological adaptations provide valuable insights into the timing and induction period of adaptations. Specifically, Pentidis et al. (46) assessed the muscle morphology in preadolescent gymnasts and untrained peers at three-month intervals over a year, providing time-course data on muscle morphological adaptations during this age group. However, due to the design limitations of other studies, a time-course analysis was not feasible.

Among all studies included herein, only 20.7% determined their sample size using an *a priori* power analysis [three cross-sectional studies (34, 36, 45), one longitudinal study (46), and two interventional studies (35, 43)]. An *a priori* power analysis is a critical procedure for sample size determination (57). This scoping review highlights the importance of building evidence in this field using appropriately determined sample sizes.

### 4.2 Population of the participants

In addition to study design and methodological rigor, the selection of the study population is also essential. The following distribution was obtained after categorizing the included studies according to participant age: aged 6–11 years, 12 articles; aged 12–14 years, 10 articles; and aged 15–17 years, 10 articles. Additionally, 34.5% of the studies did not report biological age. Testosterone (58) or insulin-like growth factor-1 (59), which influences muscle morphological adaptations, fluctuates across developmental stages. Therefore, understanding how muscles adapt to RT and sports participation at different ages and developmental stages is essential. A suitable approach for investigating age- or growth-related differences in adaptation involves designing experiments that include multiple age groups or biological ages as independent variables. This approach enables the examination of how muscle morphology adapts to the same training or sports participation across different ages or biological age groups within the same study. Our findings revealed that only two observational studies (20, 30) and one interventional study (19) adopted this experimental design. Furthermore, studies that target groups subdivided by biological maturation (e.g., early, mid, and late adolescence) would be valuable. Such study designs could provide more detailed information on muscle morphological adaptations across biological maturation.

Sex differences exist in the hormonal regulation of muscle morphology and hypertrophy (59). Extensive research has been conducted on how sex differences influence the effects of RT on muscle morphology in adults (60, 61). Among the included studies, the sex distribution was as follows: male, 13 articles; female, seven articles; both, eight articles; not listed, one article. Notably, only four observational studies (33, 34, 38, 41) and one interventional study (19) designed experiments using sex as an independent variable. For example, Gomez-Bruton et al. (34) compared the muscle CSA between young male and female swimmers. To gain a deeper understanding of muscle morphological adaptations to exercise stimuli and develop appropriate training programs, future studies should more thoroughly assess sex differences in these adaptations. Moreover, it should be noted that the number of studies involving female participants was approximately half that of those involving males (Figure 3).

### 4.3 Targeted activities

Several studies have investigated the effects of training parameters (intensity, frequency, duration, and volume) of RT on hypertrophic outcomes in adults (16, 62). Among the nine interventional studies included herein, the intervention duration ranged from 5 weeks to 10 months, with 77.8% implementing interventions lasting 12 weeks or less. The total number of interventions ranged from 14 to 60. Regarding load, various methods were employed; for example, body-mass based squat and isometric training of elbow flexion. However, none of the included interventional studies examined the influence of these factors on muscle hypertrophy. While training intensity, frequency, duration, and volume are critical components in the designing of RT programs, current evidence on muscle hypertrophy in children and adolescents remains insufficient to evaluate their specific effects.

Across the included studies, 14 different sports were analyzed for their effects on muscle morphology. Four studies combined players from various sports (Table 1). Although participants in each study had engaged in the target sports for a certain period, there were variations in the participants’ reported competitive levels. Both Hoshikawa et al. (39) and Giraldo García et al. (33) required junior soccer players as participants. In the study by Hoshikawa et al. (39), participants took part in regional and national junior competitive meets during the research period. In contrast, Giraldo García et al. (33) did not report the competitive level of their participants. Competitive level may be associated with the nature of training, which can, in turn, influence muscle morphological adaptations. Therefore, future studies should provide as much detail as possible regarding participants’ competitive level to better understand how sports participation affects muscle morphology in children and adolescents.

### 4.4 Targeted muscles and measurement methods

Not only the type of activity but also the targeted muscles and measurement methods can influence the morphological outcomes of RT and sports participation. The included studies examined the size of 47 different muscles or muscle groups (Table 2). Our results identified unexplored muscles and measurement methods. For example, the included studies did not examine the volume of lower leg muscles, which play a crucial role in human locomotion—such as walking and running—as well as in sports activities. Previous studies on adults have reported that muscle adaptations to RT (63) and sports participation (64) vary according to muscle. Abe et al. (63) reported that upper-body muscle thickness increased more rapidly and to a greater extent than lower-extremity muscle thickness. Although these findings have not been corroborated in children and adolescents, if similar heterogeneity exists in muscle adaptations, the choice of the target muscle could alter the results of muscle size adaptations. Future studies should determine whether morphological adaptations vary across the muscles.

Regarding the number of studies classified according to the measurement method, those assessing muscle volume were the fewest (Figure 4). Measuring muscle volume requires a relatively greater number of slices compared with measuring CSA or thickness, which may have limited growth in the number of such studies. However, for example, muscle volume has been shown to be more appropriate than anatomical CSA for evaluating the size–strength relationship (65), highlighting the importance of measuring muscle volume. Future research should therefore assess muscle volume to better understand the effects of RT and sports participation in children and adolescents.

### 4.5 Practical implications

This review has practical implications. First, we identified relatively fewer interventional studies than observational studies, indicating that information on the effects of RT on muscle morphology in children and adolescents remains limited for coaches, physical education teachers, and strength and conditioning professionals. Second, as discussed above, the relationships between RT and sports participation and muscle morphological adaptation in children and adolescents can be influenced by various factors such as developmental stage, sex, and the specific muscles targeted. These factors varied greatly across the studies included in this review. Moreover, in the included interventional studies, training parameters, such as intensity, frequency, duration, and volume, also varied considerably. Therefore, when those engaged in coaching and physical education attempt to apply existing evidence in practice, careful attention should be paid not only to the results and conclusions, but also to how these influential factors were defined in the original studies, as they may affect muscle morphological outcomes.

### 4.6 Limitations

This review only included studies that directly measured muscle morphology, excluding articles that reported body mass index or circumference or fat-free mass as indicators of muscle mass or size. This should be considered when interpreting the results of the present study. Moreover, we only included studies published in English. Consequently, the scope of the collected studies and regions in which the research was conducted may have been biased.

## 5 Conclusion

Changes in muscle morphology are a key aspect of the effects of RT and sports participation. However, available knowledge regarding this has not been systematically integrated. This study systematically collected and analyzed 29 studies to consolidate existing evidence in this field. Our findings reveal several key points. None of the included interventional studies used RCT designs following the CONSORT guidelines. Regarding sports participation, our findings highlight the lack of prospective studies. Moreover, our results showed that the number of studies involving female participants was approximately half that of those involving male participants and that the volume of lower leg muscles—which play a crucial role in human locomotion or sports activities—have not been assessed. These results indicate the future research directions in this field, including experimental design, participant selection, and targeted muscles.

## Data Availability

The original contributions presented in the study are included in the article/Supplementary Material, further inquiries can be directed to the corresponding author.
